# Role of WISP1 in Stellate Cell Migration and Liver Fibrosis

**DOI:** 10.3390/cells13191629

**Published:** 2024-09-29

**Authors:** Daniela González, Gisela Campos, Larissa Pütter, Adrian Friebel, Christian H. Holland, Leonhard Holländer, Ahmed Ghallab, Zaynab Hobloss, Maiju Myllys, Stefan Hoehme, Nadja M. Meindl-Beinker, Steven Dooley, Rosemarie Marchan, Thomas S. Weiss, Jan G. Hengstler, Patricio Godoy

**Affiliations:** 1 IfADo-Leibniz Research Centre for Working Environment and Human Factors, Technical University Dortmund, Ardeystrasse 67, 44139 Dortmund, Germany; gisecampos2014@gmail.com (G.C.); leonhard.hollaender@tu-dortmund.de (L.H.); ghallab@ifado.de (A.G.); hobloss@ifado.de (Z.H.); myllys@ifado.de (M.M.); marchan@ifado.de (R.M.); 2 Interdisciplinary Centre for Bioinformatics (IZBI) & Saxonian Incubator for Clinical Research (SIKT), University of Leipzig, Haertelstraße 16–18, 04107 Leipzig, Germany; friebel@izbi.uni-leipzig.de (A.F.); hoehme@uni-leipzig.de (S.H.); 3 Department of Forensic and Veterinary Toxicology, Faculty of Veterinary Medicine, South Valley University, Qena 83523, Egypt; 4 Department of Medicine II, Section Molecular Hepatology, Medical Faculty Mannheim, Heidelberg University, 69117 Mannheim, Germany; nadja.meindl-beinker@medma.uni-heidelberg.de (N.M.M.-B.); steven.dooley@medma.uni-heidelberg.de (S.D.); 5 Children’s University Hospital (KUNO), University Hospital Regensburg, 93053 Regensburg, Germany; thomas.weiss@ukr.de

**Keywords:** liver fibrosis, stellate cells, WISP1, collagen

## Abstract

The mechanisms underlying the remarkable capacity of the liver to regenerate are still not completely understood. Particularly, the cross-talk between cytokines and cellular components of the process is of utmost importance because they represent potential avenues for diagnostics and therapeutics. WNT1-inducible-signaling pathway protein 1 (WISP1) is a cytokine member of the CCN family, a family of proteins that play many different roles in liver pathophysiology. WISP1 also belongs to the earliest and strongest upregulated genes in mouse livers after CCl_4_ intoxication and has recently been shown to be secreted by tumor cells and to bind to type 1 collagen to cause its linearization in vitro and in tumor tissue in vivo. We show that WISP1 expression is strongly induced by TGFβ, a critical cytokine in wound healing processes. Additionally, secretion of WISP1 protein by hepatic stellate is increased in cells upon TGFβ stimulation (~seven-fold increase). Furthermore, WISP1 facilitates the migration of mouse hepatic stellate cells through collagen in vitro. However, in WISP1 knockout mice, no difference in stellate cell accumulation in damaged liver tissue and no influence on fibrosis was obtained, probably because the knockout of WISP1 was compensated by other factors in vivo.

## 1. Introduction

The extracellular matrix is a three-dimensional dynamic network that connects cells and regulates their functions [1,2]. Constituents of the extracellular matrix are collagens, laminins, fibronectin, elastin, hyaluronan, proteoglycans, and numerous matricellular proteins, which are constantly remodeled by a fine-tuned process of matrix degradation and synthesis [3,4]. The composition and structure of the extracellular matrix are markedly modified during the regeneration of acute tissue damage, progression of chronic disease, and cancer.

In the liver, the extracellular matrix is observed particularly in the perisinusoidal space, also called Disse space, which has a width that varies between 0.2 and 1 µm and is located between the basolateral side of hepatocytes and the fenestrated sinusoidal endothelial cells (LSECs). The Disse space contains the hepatic stellate cells (HSCs) that face sinusoidal endothelial cells and also the hepatocytes, interposed by extracellular matrix. Upon liver damage, stellate cells accumulate in the necrotic area by migration and proliferation and contribute to the pathogenesis of fibrosis [5,6,7]. To be able to migrate through the extracellular matrix, HSCs have been reported to produce matrix-degrading enzymes, such as matrix metalloproteinases (MMPs), but the exact mechanism that enables HSC mobility has not yet been sufficiently elucidated [8,9].

Recently, the matricellular protein WISP1 has been shown to be secreted by tumor cells and to bind to type 1 collagen to cause its linearization in vitro and in tumor tissue in vivo [10]. Linearization of collagen in tumor tissue of mice did not influence the growth of the primary tumors but significantly increased the number of distant metastases [10]. WISP1 has also been linked to processes such as cell death, extracellular matrix production, cell migration, and proliferation [11] and to chronic liver diseases such as fibrosis and hepatocellular carcinoma development [12,13,14].

In the present study, we observed that HSCs secrete WISP1 protein in response to liver damage and TGFβ exposure. Therefore, we studied whether WISP1 facilitates the migration of HSCs through collagen and if this process is relevant for acute and chronic liver damage in WISP1 knockout mice.

## 2. Materials and Methods

### 2.1. Animal Experiments

All animals, BALB/cJRj (Janvier Labs, Le Genest-Saint-Isle, France) and WISP1 wild-type and full knockout mice (Lexikon Pharmaceuticals, The Woodlands, TX, USA), were kept and treated according to animal welfare and European laboratory animal regulations. Mice had free access to water and food (Ssniff R/M-H, 10 mm standard diet, Ssniff, Soest, Germany), the room temperature was kept between 18 °C and 26 °C, the humidity was maintained between 30% and 70%, and a 12-h light/dark cycle was used. As a model of acute liver injury, the hepatotoxic compound acetaminophen was used. Mice were starved overnight, and a single dose of 300 mg/kg paracetamol (Sigma Aldrich, St. Louis, MO, USA), dissolved in sterile 1x PBS, was administered intraperitoneally [15]. To study the role of WISP1 in the pathology of liver fibrosis, a dose of 1 g/kg CCl_4_ (Sigma Aldrich, St. Louis, MO, USA) dissolved in olive oil was administrated intraperitoneally (i.p.) twice a week for 2 months. Blood sampling and organ collection were conducted as described previously [16].

### 2.2. Cell Isolation and Cell Culture

Primary mouse liver cells were obtained from male BALB/cJRj mice using a two-step perfusion or a three-step perfusion technique and cultured as described previously [17]. Hepatocytes were further purified by one Percoll (GE Healthcare, Düsseldorf, Germany) centrifugation step. LSECs and KCs were isolated using the magnetic-activated cell sorting (MACS) method [18] using an autoMACS Pro Separator according to the manufacturer’s instructions (Miltenyi Biotec, Bergisch Gladbach, Germany) CD146 MicroBeads (Miltenyi, Bergisch Gladbach, Germany) were used to isolate LSECs and anti-F4/80 MicroBeads UltraPure (Miltenyi, Bergisch Gladbach, Germany) to isolate KCs. Hepatic stellate cells (HSCs) were isolated as described previously [17]. Cells were maintained in DMEM medium plus additives and 10% sera plus at 37 °C and 5% CO_2_. Stimulation with TGFβ (10 ng/mL) of primary mouse hepatocytes, LSECs, KCs, and HSCs was performed for 48 h, and supernatants were collected and frozen or directly used for protein analysis.

Stimulation of stellate cells with recombinant WISP1 (Antibodies Online, Aachen, Germany) at a concentration of 25 µg/mL was performed for 24 h after 16 h of starvation in DMEM without serum on plastic cell culture dishes. At the end of the stimulation period, RNA was collected for gene expression analysis.

### 2.3. Migration Assay

Cell migration and invasion were studied using a trans-well assay. Collagen solutions with and without recombinant WISP1 (Antibodies Online, Aachen, Germany) were prepared as described previously [10]. Briefly, bovine collagen I (PureCol I, BioMatrix. San Diego, CA, USA) was neutralized to pH 7.0 on ice by adding 0.1 M NaOH and mixed with 10 x PBS to yield a stock solution containing 2 mg/mL of collagen in 1 x PBS. Recombinant WISP1 (Antibodies online, Aachen, Germany) in a concentration of 200 μg/mL and bovine serum albumin 200 μg/mL were used to prepare the different collagen solutions. Stellate cells in serum-free media were seeded on collagen-coated 24-well trans-well inserts membranes (Corning) and incubated for 20 min at 37 °C. Afterward, serum-containing media was added to the well. After 72 h, the cells were stained and fixed with 0.5 *w*/*v* Crystal Violet in methanol/water. Inserts were then washed thrice with tap water and dried overnight. Finally, cells that invaded the bottom chamber were imaged with a Primovert Zeiss bright-field microscope and later quantified with Image J V 1.8.0 (Bethesda, MD, USA).

### 2.4. Collagen and WISP1 Protein Quantification

Quantification of collagen was performed using the Sircol collagen kit (Biocolor, Belfast, UK) following the manufacturer’s instructions. Briefly, collagen was washed three times with 1 x PBS, following overnight incubation with 0.5 M cold acetic acid at 4 °C. To extract and concentrate the collagen, samples were treated with an acid-neutralizing reagent and an isolation and concentration reagent. After overnight incubation at 4 °C and centrifugation, the hydrated transparent collagen pellets were re-suspended in acetic acid and the Syrcol Dye. Finally, absorbance at 555 nm was measured using a microplate reader (Infinite M200 Pro), and concentrations of collagen were calculated according to the standard curve and manufacturer’s instructions. Quantification of WISP1 protein was conducted using the Mouse/Rat WISP-1/CCN4 Quantikine ELISA kit (R&D Systems, Minneapolis, MN, USA) according to the manufacturer’s protocol.

### 2.5. Histopathology and Immunohistochemistry

Four µm-thick PFA (4%)-fixed paraffin-embedded liver tissue sections were used for all staining. For histopathology analysis, hematoxylin and eosin staining were performed. For immunohistochemistry, the following primary antibodies were used: anti-desmin (RB-9014p0, Thermo Fisher Scientific, Darmstadt, Germany; dilution 1:400) and anti-α-SMA (ab150301, abcam, Cambridge, UK; dilution 1:100). Whole slide scans were acquired with Axio Scan.Z1, Zeiss (Jena, Germany).

### 2.6. Image Analysis

Bright-field whole slide scans were used to analyze tissue and Sirius red-positive regions. Segmentations were conducted with QuPath [19] in WISP1 KO and WT mice 2 months after CCl_4_ treatment. A random forest-based pixel classifier was interactively trained for tissue segmentation, excluding the vein lumen, at a resolution of 3.54 μm, and for Sirius red segmentation at 0.44 μm. During post-processing, performed at a pixel resolution of 0.44 μm, the tissue mask was eroded to remove a 30 μm-wide margin in order to exclude the normal collagen deposition in large blood vessels. The resulting fibrosis segmentation was skeletonized with the Python library scikit-image [20] using Lee’s method [21]. Three measurements were obtained: ‘Fibrosis fraction’ refers to the proportion of fibrosis area to the total tissue area, ‘length density’ is the length of the skeleton normalized to the area of the total tissue, and ‘compactness’, which is defined as the area of the fibrosis segmentation divided by its perimeter.

Total and activated stellate cells in relation to dead cell areas were analyzed in bright-field whole slide scans. Random forest pixel classifiers were interactively trained for segmentation of tissue at 3.54 μm, pericentral dead cell areas at 1.77 μm, and desmin/alpha-SMA signal at 0.44 μm using QuPath. Dead cell areas were not in all images clearly distinguishable or not detectable (nd). No additional post-processing was applied. The segmented desmin/alpha-SMA positive areas within the dead cell regions were quantified in terms of their area, normalized by the area of the necrotic regions.

### 2.7. Analysis of Blood Biomarkers of Liver Damage

Biomarkers of liver damage in mouse plasma were measured using the Piccolo Xpress Chemistry Analyzer (Hitado, Möhnesee, Germany). Plasma samples were diluted 1:1 with PBS.

### 2.8. RNA Isolation, cDNA Synthesis and Real-Time Quantitative PCR

RNA was isolated from mouse liver or plated cells and real-time quantitative PCR was performed as described previously [22]. Taqman primer probes (Applied Biosystems, Darmstadt, Germany) are described in Supplementary Table S1. PCR analysis of WISP1 mRNA was performed in liver tissue after CCl_4_ intoxication and in mouse hepatocytes stimulated with TGFβ in vitro. Primers were designed, flanking the un-spliced transcript (full-length variant of WISP1) of about 800 bp.

### 2.9. Western Blot

Western blot analysis was conducted as described previously [22]. Immunoblot detection of WISP1 in supernatants from primary mouse hepatocytes stimulated with 10 ng/mL TGFβ for 48 h and transfected with either scrambled (Scr) or WISP1 siRNA oligonucleotides (siWISP1) was performed using Sheep anti-WISP1 (R&D Systems, Minneapolis, MN, USA) 1:500 in 5% BSA. Protein detection was performed by chemiluminescence using a Fusion-FX7 imager (Vilber Lourmat, Eberhardzell, Germany).

### 2.10. Statistical Analysis

Data were analyzed using Prism software (GraphPad Prism 9.5.0 Software, Inc., La Jolla, CA, USA). The applied statistical tests are indicated in the respective figure legends.

## 3. Results

### 3.1. WISP1 Is Secreted by Hepatocytes and Stellate Cells in Response to Acute Liver Damage and TGFβ

**Figure 1:**
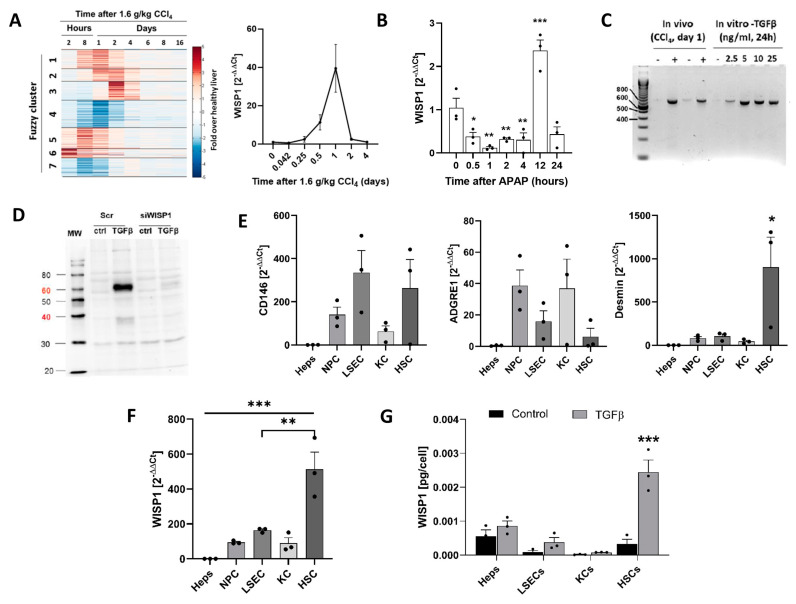
Expression and secretion of WNT1-inducible-signaling pathway protein 1 (WISP1) by hepatocytes and non-parenchymal cell types. (**A**) Gene array data of mice at various time periods after administration of CCl_4_ (1.6 g/kg) and confirmation of WISP1 mRNA levels by qRT-PCR. The individual fuzzy clusters contain genes with a similar time course after the administration of CCl_4_. This heatmap is adapted from a previous publication. Data are means ± SE of three mice per time point. (**B**) Induction of WISP1 RNA levels by an overdose of paracetamol (APAP; 300 mg/kg). Data are means ± SE of three mice per time point. (**C**) PCR analysis of WISP1 mRNA in mouse liver after CCl_4_ administration or in primary hepatocytes after TGFβ stimulation with the indicated concentrations for 24 h. The primers used were designed to flank the full-length transcript of WISP1. (**D**) Verification of the specificity of the WISP1 antibody by siRNA-mediated knockdown in cultured hepatocytes. Data represent three independent experiments. (**E**) Enrichment of liver sinusoidal endothelial cells (LSECs), Kupffer cells (KCs), hepatic stellate cells (HSCs), hepatocytes (HEP), and non-parenchymal cells (NPCs); (mixed) and characterization by the markers CD146, ADGRE1 and desmin (qRT-PCR). Data are means ± SE of three independent experiments. (**F**) Expression (qRT-PCR) and (**G**) secretion (ELISA) of WISP1 by cultured hepatocytes (Heps), LSECs, KCs, and HSCs (48 h) with and without stimulation by TGFβ. Data are means ± SE of three independent experiments. * *p* < 0.05; ** *p* < 0.01; *** *p* < 0.001 to control, unpaired *t*-test.

In a previously published genome-wide expression study of the livers of mice at several time periods after a hepatotoxic dose (1.6 g/kg) of CCI_4_, WNT 1-inducible Signaling Pathway Protein 1 (WISP1, synonym: CCN4) attracted our attention because it belongs to the earliest and strongest upregulated genes (Fuzzy cluster 1) (Figure 1A). WISP1 expression increased already at the earliest analyzed time point of 2 h, reached a maximum at 24 h, and returned to control levels ~2 days after intoxication. Similarly, a data set with mouse livers after a single APAP overdose (300 mg/kg) showed a transient transcriptional increase in WISP1 (Figure 1B). PCR analysis of WISP1 mRNA in liver tissue from mice 24 h after CCI_4_ (1.6 g/kg) intoxication revealed a single product of about 800 bp, which corresponds to the full-length variant of WISP1 (Figure 1C). Immunoblot analysis of WISP1 in the culture medium supernatant of the primary mouse hepatocytes showed that TGFβ induced the secretion of WISP1 protein (Figure 1D). The specificity of the WISP1 antibody was verified by knockdown with WISP1 siRNA oligonucleotides (Figure 1D).

To study if only hepatocytes or also other cell types of the liver express and secrete WISP1, we separated individual NPC types, specifically liver sinusoidal endothelial cells (LSECs), Kupffer cells (KCs), and hepatic stellate cells (HSCs) and verified their identity by the markers CD146, ADGRE1 and desmin (Figure 1E). Interestingly, HSCs showed the highest levels of WISP1 mRNA, which clearly exceeded the levels of hepatocytes, LSECs, KCs, and the mixed NPC population (Figure 1F). Analysis of the culture medium supernatant for WISP1 by ELISA showed that both hepatocytes and HSCs secreted WISP1 protein in an order of magnitude of 5.6 × 10^−^^4^ and 3.36 × 10^−^^4^ pg/cell in 48 h, respectively. (Figure 1G). Stimulation by TGFβ (10 ng/mL) caused the strongest approximately seven-fold increase in WISP1 secretion in HSCs. Hepatocytes showed relatively high basal levels but only a relatively small induction by TGFβ.

### 3.2. WISP1 Stimulates the Migration of Stellate Cells through Collagen In Vitro

**Figure 2:**
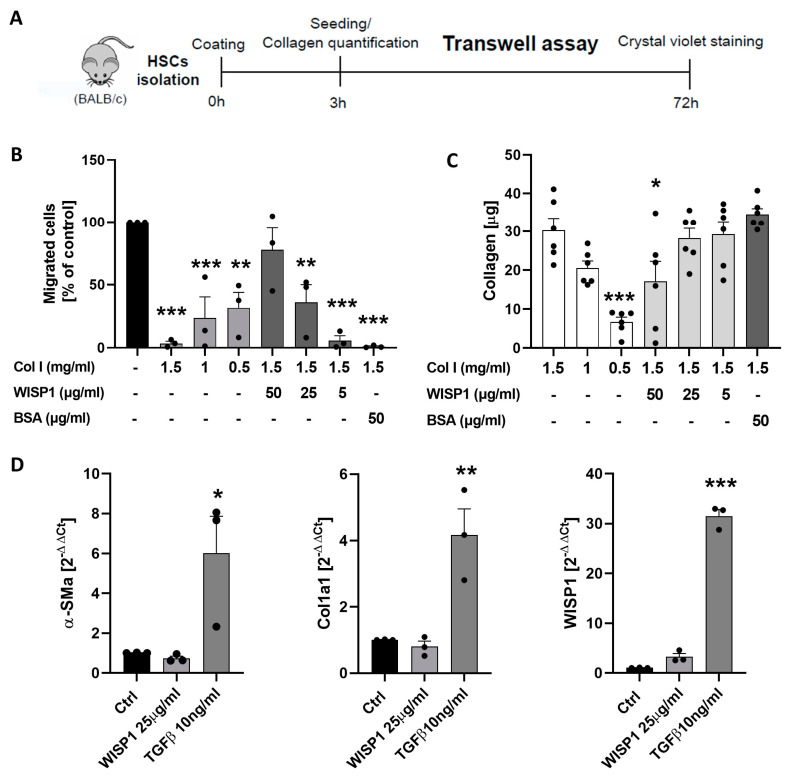
WNT1-inducible-signaling pathway protein 1 (WISP1) influences the migration of stellate cells through collagen in vitro. (**A**) Experimental design. (**B**) Influence of collagen and WISP1 on the passage of hepatic stellate cells (HSCs) through the membrane. (**C**) Influence of WISP1 on the amount of collagen on membranes. (**D**) Influence of WISP1 and TGFβ (positive control) on the expression of α-SMA, Col1a1, and WISP1. The data are presented as means ± standard errors of 3 biological replicates. * *p* < 0.05; ** *p* < 0.01; *** *p* < 0.001 to control, one-way ANOVA. Compared to the control with 1.5 mg/mL Col I, BSA (50 µg/mL), without WISP1, the sample with 50 µg/mL of WISP1 shows significantly higher migration (*p* < 0.01).

Since acute liver damage and exposure to TGFβ stimulated the expression and secretion of WISP1 by stellate cells, we next focused on possible functional consequences. Recently, WISP1 has been reported to linearize collagen and increase its permeability to migrating tumor cells [10]. To study if a similar effect is observed for stellate cells, we used a trans-well chamber where the migration of HSCs through a membrane was induced by serum as a chemoattractant (Figure 2A). The membrane was coated with collagen type I without and with the addition of several concentrations of recombinant human WISP1. Compared to uncoated membranes, collagen at concentrations of 1.5, 1.0, and 0.5 mg/mL strongly reduced the number of stellate cells that passed through the membrane (Figure 2B). For example, 1.5 mg/mL of collagen reduced migration to 3.49% as a percentage of the control (uncoated membranes). Importantly, the addition of recombinant WISP1 to collagen (1.5 mg/mL) at 5, 25 and 50 µg/mL concentration-dependently increased the number of HSCs that passed through the membrane (Figure 2B). Compared to the 1.5 mg/mL collagen-coated filters without WISP1 the effect of 25 and 50 µg/mL of WISP1 was statistically significant. In contrast, the addition of 50 µg/mL bovine serum albumin (BSA) as a negative control to 1.5 mg/mL of collagen did not increase HSC passage.

One possibility for how WISP1 may facilitate the migration of HSCs is the linearization of collagen, as previously reported [10]. An alternative possibility is that WISP1 may influence the stickiness of collagen. Stickiness is defined as the amount of collagen bound to the 24-well trans-well insert membranes by the coating procedure. If WISP1 influences the stickiness of collagen, different amounts of collagen will bind to the membrane, which may secondarily influence HSC migration. To test this possibility, the collagen amount on coated membranes was quantified by a colorimetric assay (Soluble collagen assay Sircol, Biocolor) after harvesting the collagen from the membrane by chemical hydrolysis using cold acetic acid 0.5 M. Indeed, 50 µg/mL of WISP1 significantly reduced the collagen amount (Figure 2C). However, 25 and 5 µg/mL of WISP1 did not reduce the collagen amount on the membranes, while these concentrations were already sufficient to enhance the passage of HSCs through collagen. Thus, WISP1 reduced the stickiness of collagen on polyethylene terephthalate (PET) membranes at high concentrations but below these concentrations it already altered the properties of collagen to facilitate the migration of HSCs.

An important question is whether WISP1—at concentrations where it alters the properties of collagen—also activates HSCs. To address this question, cultivated HSCs were exposed to WISP1 (25 µg/mL) or to TGFβ (10 ng/mL) as a positive control. While TGFβ was used as a positive control strongly enhanced the expression of α-SMA and collagen 1a1, WISP1 did not show a significant influence (Figure 2D). As already reported above, TGFβ strongly induced the expression of WISP1 in HSCs; in contrast, WISP1 at 25 µg/mL did not significantly influence its own expression (Figure 2D).

### 3.3. Acute and Chronic Liver Damage in WISP1 Knockout Mice

**Figure 3:**
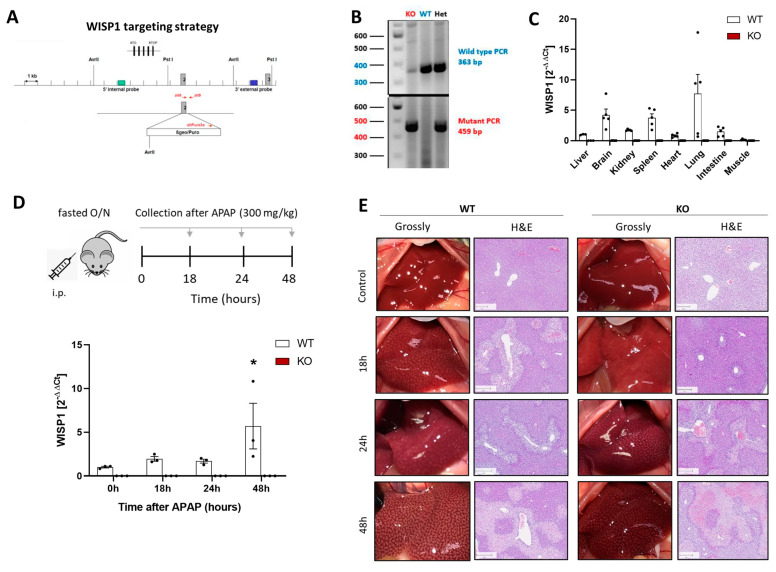
WNT1-inducible-signaling pathway protein 1 (WISP1) knockout in acute liver damage. (**A**) Knockout strategy (**B**) verification of the knockout strategy by PCR and (**C**) qRT-PCR of WISP1 in various organs. (**D**) Experimental design of acute APAP overdose and verification of the WISP1 knockout by qRT-PCR, * *p* < 0.05, one-way ANOVA. (**E**) Macroscopic appearance of the livers of WISP1 KO and WT mice as well as Hematoxylin–Eosin (H&E) stainings. Scale bars 200 µm.

The data presented in the previous paragraph suggests that WISP1 may modify collagen in the extracellular matrix to allow HSCs to migrate more efficiently. Other than several other functions, HSCs play a role after acute liver damage when they migrate into and/or are activated in damaged tissue regions and support the regeneration process. Moreover, HSCs are relevant for the pathogenesis of chronic liver disease, where they contribute to the formation of fibrotic streaks [23]. To address a possible role in acute and chronic liver damage, full-body WISP1 knockout mice were generated. For this purpose, exon 2 of WISP1 was replaced by a construct containing a puromycin cassette and a stop codon in its 3′ end region, resulting in a block of transcription (Figure 3A). Genotyping was performed by PCR using primers that generated a 363 or 459 bp product in wild-type or knockout mice, respectively, thereby allowing a clear differentiation between homozygous knockout, homozygous WT, and heterozygous mice (Figure 3B). The successful knockout was validated by qRT-PCR of several organs of WT and KO mice (Figure 3C).

To study the role of WISP1 in acute liver damage, we used an experimental design [15], where 300 mg/kg of APAP was administered and liver tissue was analyzed after 18, 24 and 48 h (Figure 3D). In WT mice, an APAP overdose led to increased WISP1 expression, whereas WISP1 was below detection limit in all KO mice (Figure 3D). The APAP-induced pericentral liver damage was macroscopically visible as white dots and as pale regions in hematoxylin and eosin-stained sections (Figure 3E).

**Figure 4:**
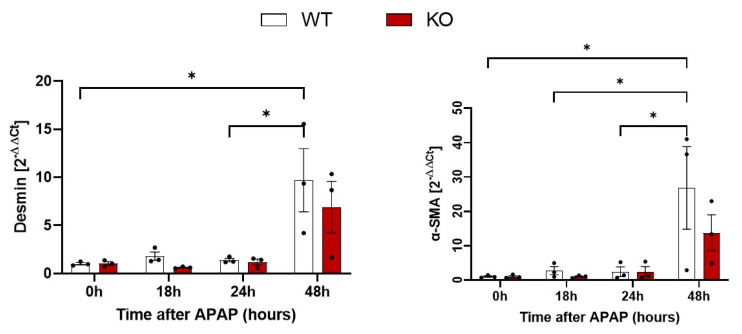
Quantification of desmin and α-SMA positive cells after administration of a hepatotoxic dose of APAP. qRT-PCR of desmin and α-SMA mRNA levels in liver tissue after APAP overdose. The data are presented as means ± standard errors of three biological replicates. * *p* < 0.05; compared to control, one-way ANOVA.

**Figure 5:**
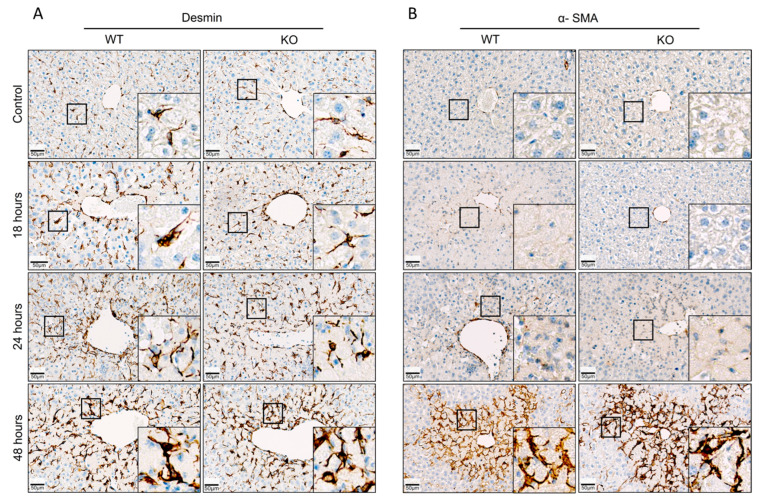
(**A**) Visualization of quiescent and activated hepatic stellate cells (HSCs) 18, 24 and 48 h after administration of a hepatotoxic dose of acetaminophen (APAP). (**A**) Immunostaining of the quiescent HSC maker desmin. (**B**) Immunostaining of α-SMA, a marker of activated HSCs.

To quantify a possible difference between WT and KO mice, desmin and α-SMA in liver tissue were quantified by qRT-PCR (Figure 4). An increase of both desmin and α-SMA RNA was observed, particularly at 48 h after APAP overdose. However, no significant difference between WT and KO animals was obtained at any of the analyzed time periods (Figure 4). Next, immunostaining of desmin and α-SMA was performed to visualize total and activated HSCs, respectively (Figure 5). Desmin-positive HSCs appeared as longish cells in both control liver tissue and after APAP overdose (Figure 5A). In contrast, α-SMA-positive HSCs were not observed in the liver tissue of controls but occurred after APAP overdose, particularly in the pericentral dead cell areas 48 h after APAP overdose (Figure 5B). In agreement with the RNA results, no obvious differences were seen between the immunostained sections of WT and Wisp1 KO mice. Together, these results speak against a major difference concerning the activation of stellate cells and their presence in the dead cell area between WT and Wisp1 KO mice.

**Figure 6:**
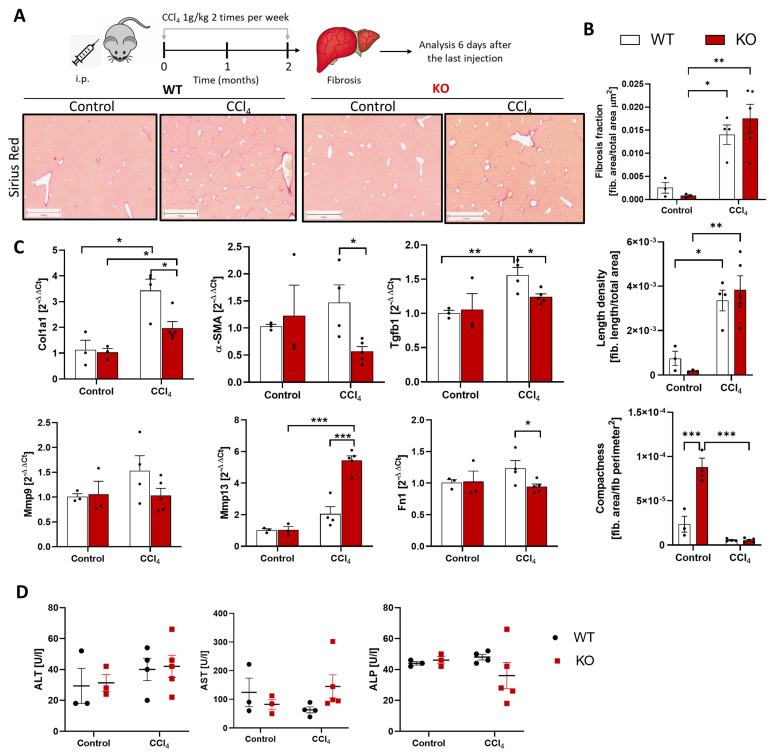
WNT1-inducible-signaling pathway protein 1 (WISP1) knockout in chronic liver damage. (**A**) Experimental schedule of CCl_4_ induced fibrosis and representative examples of fibrotic streaks. Scale bars 1000 µm (**B**) Image analysis and quantification of fibrotic streaks. Data are presented as means ± standard errors of at least three mice per group. * *p* < 0.05; ** *p* < 0.01; *** *p* < 0.001, two-way ANOVA. (**C**) Expression of fibrosis-associated genes. Results are plotted as a log-fold increase compared to the control liver and are presented as means ± standard errors of at least three mice per group. * *p* < 0.05; ** *p* < 0.01; *** *p* < 0.001, multiple *t*-test. (**D**) Activity of ALT, AST, and ALT in blood.

Next, we analyzed if the WISP1 knockout influences the formation of fibrotic streaks in chronic liver disease. For this purpose, mice were treated with CCI_4_ twice a week for 2 months, and analyses were performed 6 days after the last injection (Figure 6A). Fibrotic streaks were stained by Sirius red, and image analysis was performed based on whole slide scans. No significant differences between CCl_4_-treated WT and KO mice were obtained for the area ratio, defined as the ratio of the Sirius red-positive area to the total analyzed area; the length density, defined as the total length of fibrotic streaks per area; and the compactness, defined as Sirius red-positive area per square perimeter of fibrotic streaks (Figure 6B). The differences between untreated WT and KO mice should be interpreted with caution, since the Sirius red signal was very low in these mice. While the WISP1 knockout did not influence the extent and structure of fibrosis, expression of some fibrosis-associated genes, such as Col1a1, Fn1, Tgfb1, and Mmp13 showed significant differences between WT and KO mice after CCI_4_ exposure (Figure 6C). The activity of the liver enzymes ALT, AST, and ALP in blood did not differ significantly between WT and KO mice (Figure 6D).

**Figure 7:**
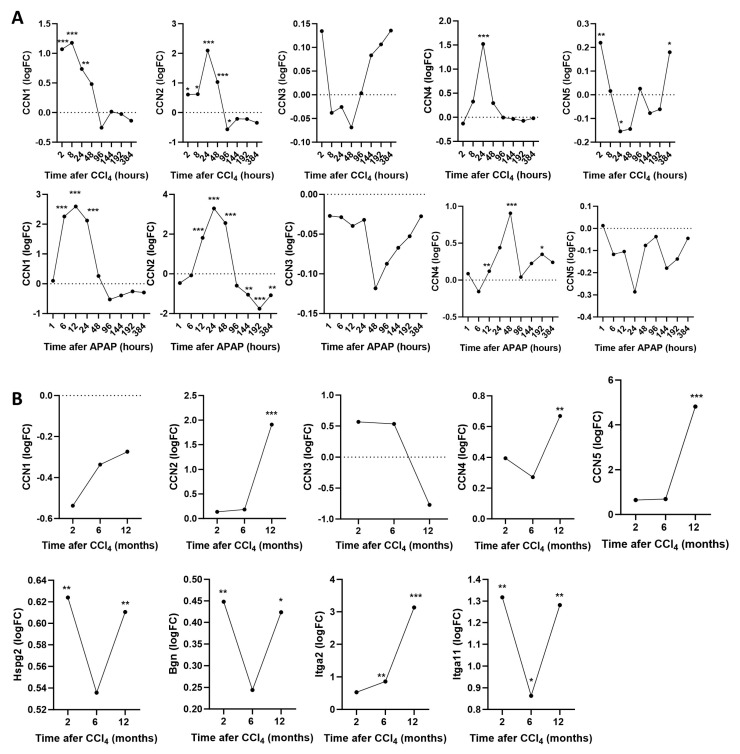
Expression of the CCN (connective tissue growth factor, cysteine-rich protein, and nephroblastoma overexpressed) gene family and further collagen-binding proteins during acute and chronic exposure to CCl_4_ (**A**) Expression of CCN proteins after single doses of CCl_4_ and APAP. (**B**) Expression of CCN proteins and collagen-binding proteins during chronic CCl_4_ exposure. * *p* < 0.05; ** *p* < 0.01; *** *p* < 0.001, paired *t*-test.

Since the lack of a phenotype concerning HSC migration and fibrosis in WISP knockout mice was surprising, we revisited the expression of all CCN (connective tissue growth factor, cysteine-rich protein, and nephroblastoma overexpressed gene) family members and further collagen-binding proteins based on previously published gene expression data in WT mice. The expression values of these genes were obtained from the corresponding Zenodo archive (https://zenodo.org/records/7242764, accessed on 18 July 2024) and have been computed previously [24]. We observed that, other than CCN4 (WISP1), CCN1 and CCN2 (CTGF) also increased after single doses of CCl_4_ and APAP (Figure 7A). After chronic CCl_4_ intoxication CCN2 (CTGF) and CCN5 (WISP2) strongly increased (Figure 7B). Other than the aforementioned CCN family members, the expression of other collagen-binding proteins, such as the proteoglycans perlecan and biglycan as well as the integrins Itga2 and Itga11, also significantly increased during chronic CCl_4_ exposure (Figure 7B). Thus, a complex response of several collagen-binding proteins was observed after the induction of acute and chronic liver damage. It should be considered that WISP1 is only one of several collagen-binding proteins responding to CCl_4_ intoxication.

## 4. Discussion

The matricellular protein WISP1 has been reported to bind to collagen type 1, promote its linearization, and enhance the migration and metastasis of tumor cells [10]. WISP1 binds to type 1 collagen by its cysteine-rich C-terminal domain (CT) to support linearization, while WISP2 lacks the CT domain and antagonizes the linearizing effect of WISP1 [25]. Further studies have reported that high expression of WISP1 is associated with metastasis and poor prognosis of hepatocellular cancer [26], with the lymphatic and perineural invasion of tumor cells of cholangiocarcinoma [27] and with liver fibrosis [28].

In the present study, we observed that WISP1 is strongly transcriptionally upregulated after induction of acute liver damage. Comparing isolated hepatocytes and non-parenchymal liver cells, we observed that HSCs show the strongest induction of WISP1 expression in response to liver damage in vivo and to TGFβ exposure in vitro. This motivated us to study the hypothesis that secretion of WISP1 by HSCs facilitates their migration through the extracellular matrix upon liver damage and supports the accumulation of HSCs in damaged tissue as well as the formation of fibrosis.

The analysis of cultured HSCs demonstrated that these cells secreted WISP1 protein into the culture medium; while secretion occurred already under control conditions, it was induced by a factor of about seven by the addition of TGFβ to the culture medium. In hepatocytes, induction of WISP1 secretion by TGFβ was weaker compared to HSCs. However, considering that basal secretion was even slightly higher than that of HSCs and that the total number of hepatocytes in liver tissue is higher compared to HSCs, it can be expected that also the contribution of hepatocytes to the levels of WISP1 in liver tissue may be relevant.

To study if WISP1 alters the properties of collagen, we performed migration experiments with trans-well dishes, where primary HSCs migrate through collagen type 1, which contains different concentrations of WISP1. Indeed, WISP1 at concentrations of 25–50 µg/mL increased migration of HSCs through the collagen-coated filters. At 50 µg/mL, the properties of collagen were further altered, resulting in decreased stickiness to the polyethylene terephthalate membranes. The effect size of 25 µg/mL of WISP1 was high, considering that the percentage of HSCs that migrated through the collagen layer was increased by a factor of approximately ten compared to the BSA control. Thus, it may appear likely that secretion of WISP1 also facilitates HSC migration in vivo.

To address the role of WISP1 for HSC migration in vivo, we used constitutive knockout mice. Other than understanding the in vivo situation, the motivation of these experiments was also to learn if WISP1 may represent a therapeutic target to ameliorate HSC accumulation and fibrosis in liver disease. The knockout was efficient as evidenced by a decrease in WISP1 mRNA levels below the detection limit in all investigated organs, including the liver. However, the WISP1 knockout did not modify the accumulation of HSCs in damaged liver tissue in a time-dependent analysis after acute injury, nor did it alter the extent and architecture of fibrosis in chronic scenarios. Although this lack of a phenotype was unexpected, it should be considered that the structure of collagen is controlled by numerous factors that may compensate for each other [29,30]. For example, expression of matrix metalloprotease 13 (MMP13) was similar in untreated WISP1 KO and WT mice but increased to three-fold higher levels in KO than WT mice after chronic CCl_4_ treatment. Similar to other metalloproteases, MMP 13 participates in collagen degradation [31]. Additionally, other fibrosis-associated genes such as TGFβ, Col1a1, α-SMA, and Fn1 showed lower expression levels in WISP1 knockout mice. It should also be considered that other than WISP1 (CCN4), other CCN family members such as CCN2 (CTGF) and CCN5 (WISP2), were upregulated during chronic CCl_4_ treatment. Moreover, several collagen-binding proteins increased in the chronic CCl_4_ mouse model, including perlecan, Hga2, Hga11, and biglycan. Therefore, numerous factors may compensate for the lack of WISP1 in vivo.

## 5. Conclusions

In conclusion, WISP1 is secreted by HSCs and facilitates their migration through collagen in vitro. However, no difference in HSCs accumulation in damaged liver tissue and no influence on fibrosis was obtained in WISP1 knockout mice, probably because the knockout of WISP1 was compensated by other factors. Therefore, WISP1 antagonizing therapies do not appear to represent a promising therapeutic strategy in liver fibrosis.

## Data Availability

The data presented in this study are available on request from the corresponding author.

## Supplementary Materials

The following supporting information can be downloaded at: https://www.mdpi.com/article/10.3390/cells13191629/s1, Table S1: qPCR Taqman primer probes.

## Abbreviations

ALT	alanine transaminase
AST	aspartate transaminase
ALP	alkaline phosphatase
i.p.	intraperitoneal
Heps	hepatocytes
NPC	non-parenchymal cells
KCs	Kupffer cells
LSECs	liver sinusoidal endothelial cells
HSCs	hepatic stellate cells
TGFβ	tumor growth factor beta
